# Therapierefraktäre erstmalige Depression im mittleren Lebensalter

**DOI:** 10.1007/s00115-023-01530-3

**Published:** 2023-08-17

**Authors:** Alexander Grotemeyer, Johannes Kornhuber, Philipp Spitzer

**Affiliations:** Psychiatrische und Psychotherapeutische Klinik, Universitätsklinikum Erlangen, Schwabachanlage 6, 91054 Erlangen, Deutschland

## Hintergrund

Depressive Syndrome sowie Angsterkrankungen sind sehr häufige psychische Erkrankungen, die in aller Regel gut behandelbar sind. Besondere Aufmerksamkeit ist jedoch erforderlich, wenn eine depressive Episode erstmalig im höheren Lebensalter auftritt, eine medikamentöse und psychotherapeutische Therapie nicht anschlägt und begleitende neurologische Symptome feststellbar sind. Beide hier dargestellten Fälle zeigen, dass die kontinuierliche kritische Prüfung von Vordiagnosen und initialen Annahmen für Betroffene und Behandlungsteam von entscheidender Bedeutung sein kann.

## Fallbeschreibung Fall 1

Eine 58-jährige Patientin stellt sich mit einer seit aktuell 2 Jahren andauernden unipolar depressiven Episode im Rahmen einer erstmals im Alter von 52 Jahren diagnostizierten rezidivierenden depressiven Störung vor. Seit 2 Jahren bestünde eine deutlich gedrückte Stimmung. Sie habe die Freude an Tätigkeiten verloren, die sie sonst gerne gemacht habe, und sei seit 2 Jahren nicht mehr in der Lage, ihren Beruf auszuüben. Es fanden sich ein reduzierter Antrieb und vermehrte Ermüdbarkeit. Das Selbstvertrauen zeigte sich deutlich herabgesetzt bei zeitweise auftretenden Suizidgedanken ohne klare Handlungsabsichten (positive Familienanamnese für Suizid). Eine Ich-Störung oder Wahnideen fanden sich nicht. Neben einer kombinierten Ein‑/Durchschlafstörung fand sich auch ein deutlich reduziertes kognitives Leistungsniveau. Im Vorfeld wurde bereits die Diagnose einer Pseudodemenz im Rahmen einer Demenzabklärung gestellt (Mini-Mental-Status-Test (MMST) 14/30 Punkte, Montreal-Cognitive-Assessment (MoCA) 12/30 Punkte). Hierbei fand sich in der Liquordiagnostik einzig ein leicht erhöhtes Phospho-Tau-Protein (54,1 pg/ml; Erlangen-Score: 1). Im Beck-Depressionsinventar (BDI)-II ergab sich bei Aufnahme eine Punktzahl von 29 Punkten (schwere Depression). Zusätzlich wurde eine leichtgradige Angstsymptomatik berichtet. Eine antidepressive Medikation mit Duloxetin (Steigerung vor 3 Monaten von 90 auf 120 mg) führte über insgesamt 12 Monate zu keiner Besserung. Ungefähr 2 Monate vor der aktuellen stationären Aufnahme wurde ein neu aufgetretenen linksbetonter Ruhe- und Haltetremor als essentieller Tremor (ET) eingeordnet und mit Propranolol (4 × 10mg/Tag) behandelt. Die ET-Symptomatik besserte sich hierunter nur bedingt. Erschwerend kam eine relevante Aggravierungstendenz der Patientin bezüglich des Tremors hinzu. Im neurologischen Untersuchungsbefund zum Zeitpunkt der Aufnahme fand sich neben dem linksbetonten Tremor und der globalen Bradykinesie, eine Dysathrophonie, ein links betonter Rigor sowie eine posturale Instabilität. Der Glabellareflex zeigte sich wie der Palmomentalreflex (bds.) disinhibiert. Pyramidenbahnzeichen fanden sich indes nicht.

Als Medikation erhielt die Patientin bei Aufnahme Pregabalin (150 mg/Tag), Duloxetin (120 mg/Tag), Quetiapin retard (50 mg z. N.) und Propranolol 40 mg/Tag. Im Labor zeigte sich ein CRP von 3,6 mg/l (< 5 mg/l) bei Aufnahme. Nebenbefundlich gab die Patientin deutliche präprandiale Schmerzen an.

## Therapie, Diagnostik und weiterer Verlauf Fall 1

Eine im Vorfeld erfolgte Bildgebung zeigte sich unauffällig. Der Levodopa(L-DOPA)-Test war positiv (200 mg L-DOPA; Unified Parkinson’s Disease Rating Scale (UPDRS) III von 31 auf 10 Punkte). L‑DOPA plus Benserazid 62,5 mg 1–1–1 wurde von der Patientin gut vertragen. Die pseudodemenzielle Symptomatik zeigte sich im Verlauf der Therapie rückläufig und die Stimmung hellte sich 5 Wochen nach Therapiebeginn – unter begleitender Psychotherapie – deutlich auf. Nach 7 Wochen erfolgte die Entlassung mit geringer depressiver Restsymptomatik. Eine Dopamintransporter-Szintigraphie (DAT-SPECT; I-123-FP-CIT) ergab eine signifikant reduzierte präsynpatische Dopaminrezeptordichte im Bereich des rechtsseitigen Kaudatums (Z-Score −2,2) und Striatums (Z-Score −2,0; Cut-off < −2).

## Fallbeschreibung Fall 2

Ein 66-jähriger Patient stellte sich in Begleitung seiner Ehefrau zur stationären psychiatrischen Behandlung nach einer 2 Monate zuvor erfolgten stationär-psychiatrischen Therapie vor. Seit 3 Jahren habe eine kontinuierliche und nach Angaben der Ehefrau zunehmende depressive Symptomatik bestanden. Der Patient zeigte sich im Kontaktverhalten nicht erreichbar und die Merkfähigkeit war subjektiv reduziert. Im formalen Denken war der Patient verlangsamt und gehemmt, bei i. W. regelrechtem inhaltlichem Denken. Ein Gefühl der Gefühllosigkeit konnte exploriert werden. Der Antrieb zeigte sich gehemmt und die Psychomotorik reduziert, bei begleitendem Morgentief und vermehrtem Schlafbedürfnis. Nach einem (zweiten) Suizidversuch 3 Monate zuvor war im Rahmen eines stationären Aufenthaltes die Medikation von Opipramol 150 mg/Tag und Agomelatin 25 mg/Tag auf Bupropion 300 mg/Tag und Mirtazapin 15 mg/Tag umgestellt worden, allerdings ohne dass sich der gewünschte Erfolg einstellte. Ein erstmaliger Suizidversuch lag 24 Monate zurück. In den Angehörigengesprächen wurden regressive Tendenzen des Patienten deutlich und er berichtete, das Haus seit 12 Monaten nicht mehr oder nur unter hoher Anspannung verlassen zu haben. Er gab dabei an, dass es ihm unwohl sei von den Nachbarn gesehen zu werden. Genauere Angaben waren weder von der Ehefrau noch vom Patienten selbst diesbezüglich zu erhalten. Auffällig waren weiterhin eine Amimie und eine Affektstarre. In der grob orientierenden neurologischen Untersuchung zeigte sich ein diskreter rechtsseitiger Ruhetremor, welcher vom Patienten bagatellisiert und zunächst als Zeichen ängstlicher Anspannung gedeutet wurde. Ein MoCA-Testung 2 Monate zuvor ergab ein leichtes kognitives Defizit (23/30 Punkte). Laborchemisch zeigte sich eine CRP-Erhöhung auf 6,2 mg/l (< 5 mg/l).

## Therapie, Diagnostik und weiterer Verlauf Fall 2

Aufgrund der starken Anspannung und des deutlichen Leidensdrucks des Patienten wurde initial eine Therapie mit Risperidon zur Augmentation der antidepressiven Therapie initiiert. Hierbei zeigte sich jedoch im kurzen zeitlichen Verlauf ein deutlich gebundeneres Gangbild mit vermindertem Mitschwingen beider oberer Extremitäten sowie eine Zunahme des rechtseitigen Tremors. Daraufhin wurde die Medikation mit Risperidon umgehend beendet und eine cMRT-Bildgebung durchgeführt. Diese zeigte einen altersentsprechenden Befund. In der neurologischen Untersuchung konnte eine linksbetonte akinetisch-rigide Symptomatik erfasst werden, welche nach ausreichendem Pausieren der Risperidonmedikation auch per UPDRS III erfasst wurde (42 Punkte). Im L‑DOPA-Test zeigte sich eine Reduktion des UPDRS-III-Scores von 42 auf 17 Punkte. Antrieb und Angstsymptomatik besserten sich innerhalb von 72 h unter Therapie mit L‑DOPA plus Benserazid 62,5 mg 1–1–1 deutlich. Nach 4 Wochen erfolgte die Vorstellung zur Nachbeurteilung in unserer Ambulanz. Hierbei zeigte sich ein dynamischer Patient, mit gebesserter akinetisch-rigider Symptomatik, einer remittierten depressiven Episode und fehlender Angstsymptomatik. Eine DAT-SPECT im Verlauf ergab zwar eine deutliche Seitendifferenz zuungunsten rechts (Z-Score links: Striatum +1,8, Kaudatum +1,7, Putamen +1,7; Z‑Score rechts: Striatum +0,3, Kaudatum +0,1, Putamen +0,6), dies wurde jedoch bei fehlender Abweichung vom Normkollektiv (Z-Scores < −2) als (noch) physiologisch bewertet.

## Diskussion

Depressive und ängstliche Syndrome gehören zu typischen nichtmotorischen Symptomen des M. Parkinson (PD) bzw. der Parkinson-Syndrome [1–3]. Im Rahmen dieser zwei Patienten, die sich in der Psychiatrie vorstellten, zeigen sich mehrere Schwierigkeiten in der Behandlung der Komorbiditäten und der Diagnose des PD. Beide Fälle haben gemeinsam, dass sich eine depressive Symptomatik erstmalig im höheren mittleren Lebensalter manifestierte und dabei therapierefraktär und primär chronisch verlief. Im ersten Fall zeigt sich in der vollständigen Erhebung des psychiatrischen und neurologischen Befundes ein Symptomkomplex aus psychiatrisch affektiver Symptomatik, posturaler Instabilität, einseitig betontem Tremor, Rigor und Akinese. Dies ist somit als höchst wahrscheinlich für ein Parkinson-Syndrom bzw. PD zu werten. In der Literatur findet sich ein sehr ähnlicher Fall und Verlauf [4]. Im zweiten Fall zeigte sich ein überwiegend akinetisch-rigides Parkinson-Syndrom. Allerdings auch mit einem rascheren Ansprechen der L‑DOPA-Medikation auf die affektive Symptomatik. Eine Klassifikation als PD erscheint zum aktuellen Zeitpunkt bei (noch) physiologischem DaT-SPECT sehr wahrscheinlich. Insbesondere, da Patienten mit einem „scan without evidence of deopamine deficiency“ (SWEDD) im Regelfall nicht auf dopaminerge Medikation ansprechen [5]. Aufgrund der hohen Fehldiagnoserate sollte der Krankheitsverlauf unbedingt regelmäßig und kritisch reevaluiert werden [6].

Für den klinischen Alltag lassen diese Fälle mehrere Schlussfolgerungen zu. Die Differenzialdiagnose von ET und tremordominantem PD erfordert eine gründliche neurologische (Verlaufs)untersuchung und im Bedarfsfall eine weiterführende Bildgebung. Die L‑DOPA-Responsivität zu untersuchen, erscheint sinnvoll [7]. Die begleitende affektive Symptomatik hilft hier vermutlich nur bedingt zur Differenzialdiagnose, da kontrovers zu diskutieren ist, wie eine Depression mit dem ET verknüpft ist [8]. Interessant wäre bei beiden oben beschriebenen Fällen eine transkranielle Sonographie (TCS) des Hirnstamms hinsichtlich Asymmetrie. Denn entsprechende Auffälligkeiten des Hirnstamms im TCS stellen einen Prädiktor für PD dar [9]. Diese kostengünstige Untersuchung erscheint insbesondere sinnvoll, bevor eine Therapie mit Antipsychotika wie bspw. Risperdion bei Patienten mit o. g. Merkmalen initiiert wird.

## „Take-home message“

- Eine erstmalige spätmanifestierende im Verlauf auf „klassische“ Antidepressiva therapierefraktäre Depression sollte an eine neurodegenerative Genese denken lassen. Hinweise auf eine (diskrete) neurologische Symptomatik sollten weiter abgeklärt werden.
- Eine Augmentation mit Antipsychotika sollte in diesen Fällen kritisch geprüft werden. Eine Hirnstammsonographie könnte hier im i. S. einer Kosten-Nutzen-Abwägung sinnvoll sein.
- Die neuropsychiatrische Gesamtbetrachtung von Patienten ist unverzichtbar.

## References

[1] Prange S, Klinger H, Laurencin C, Danaila T, Thobois S (2022). Depression in patients with parkinson’s disease: current understanding of its neurobiology and implications for treatment. Drugs Aging.

[2] Lv Q (2022). Depression in multiple system atrophy: Views on pathological, clinical and imaging aspects. Front Psychiatry.

[3] Kano O, Ikeda K, Cridebring D, Takazawa T, Yoshii Y, Iwasaki Y (2011). Neurobiology of depression and anxiety in parkinson’s disease. Adv Neurol.

[4] Giménez-Roldán S, Mateo D, Dobato JL (1997). Depressive pseudodementia in early Parkinson’s disease: lessons from a case with long-term follow-up. Neurologia.

[5] Stoessl AJ (2010). Scans without evidence of dopamine deficiency: the triumph of careful clinical assessment. Mov Disord.

[6] Rizzo G, Copetti M, Arcuti S, Martino D, Fontana A, Logroscino G (2016). Accuracy of clinical diagnosis of Parkinson disease. Neurology.

[7] Wolters A, Walter U, Wittstock M, Benecke R (2014). Asymmetric postural tremor preceding DOPA-responsive Parkinsonism – the transition disease. J Parkinsons Dis.

[8] Huey ED (2018). Self-report depressive symptoms are dissociated from tremor severity in essential tremor. Parkinsonism Relat D.

[9] Berg D (2011). Enlarged substantia nigra hyperechogenicity and risk for Parkinson disease: a 37-month 3-center study of 1847 older persons. Arch Neurol.

